# Personalia

**Published:** 1914-05-02

**Authors:** 


### Personalia.

THE following new members have joined the board of management of the Seamen's Hospital, Greenwich:—Sir George Dashwood, Bart., Mr. J. Cyril Nairne, Captain Robert Reynolds, R.N.R., and the Rev. F. J. Tackley, M.A., Vicar of Greenwich.

